# Comparison of thoracoscopy-guided thoracic paravertebral block and patient-controlled intravenous analgesia for postoperative analgesia after uniportal thoracoscopic pulmonary wedge resection: a prospective randomized controlled trial

**DOI:** 10.3389/fmed.2025.1554515

**Published:** 2025-05-15

**Authors:** Jianhui Du, Luyao Wei, Jinxian He, Xia Xu, Lihong Hu

**Affiliations:** ^1^Department of Anesthesiology, Lihuili Hospital Affiliated to Ningbo University, Ningbo, China; ^2^Department of Thoracic Surgery, Lihuili Hospital Affiliated to Ningbo University, Ningbo, China

**Keywords:** uniportal thoracoscopy, pulmonary wedge resection, thoracoscopic-guided, thoracic paravertebral block, patient-controlled intravenous analgesic, postoperative analgesia

## Abstract

**Introduction:**

Patients often experience persistent, intense pain following uniportal thoracoscopic pulmonary wedge resection (UTPWR). This pain is usually intervened with patient-controlled intravenous analgesia (PCIA) or thoracoscopic-guided thoracic paravertebral block (TG-TPB), a novel peripheral nerve block technique. Herein, we compared the analgesic effects of TG-TPB and PCIA post-UTPWR.

**Methods:**

Sixty patients allocated into two groups: T and P. Group T patients were administered TG-TPB with 20 mL 0.375% ropivacaine at the fourth intercostal plane before sealing the chest, and connected to a PCIA pump containing 0.9% sodium chloride (NaCl). Group P patients received TG-TPB with 20 mL 0.9% NaCl and were connected to a PCIA pump containing sufentanil. The Visual Analogue Scale (VAS) scores were recorded at 2, 6, 12, 24, 36, and 48 h postoperatively. Data on sufentanil consumption, number of PCIA presses, number of rescue analgesia interventions, adverse reactions (ARs), and the 15-item Quality of Recovery Scale (QoR-15) scores were also recorded within 24 h postoperatively.

**Results:**

Compared to the P group, the T group showed lower VAS scores at 2, 6, 12, and 24 h postoperatively, as well as lower sufentanil consumption levels, number of PCIA presses, number of rescue analgesia interventions, and ARs incidences within 24 h postoperatively (all *p* < 0.05). Furthermore, the T group showed higher QoR-15 scores within 24 h postoperatively than the P group (90.5 ± 7.3 vs. 76.6 ± 6.2; *p* < 0.001).

**Conclusion:**

Compared to PCIA, TG-TPB exerted a better analgesic effect post-UTPWR, with less opioid drug use, fewer ARs, and a significantly better recovery quality within 24 h postoperatively.

**Clinical trial registration:**

https://www.chictr.org.cn/, ChiCTR2000034726.

## Introduction

With advancements in endoscopic and minimally invasive technologies, thoracoscopic surgery has emerged as the mainstay surgical intervention (1–3). Compared to three-or multi-hole thoracoscopic surgery, uniportal thoracoscopic surgery offers the benefits of fewer incisions, mild trauma, light postoperative pain, and quick recovery (4). Rocco et al. first reported uniportal thoracoscopic pulmonary wedge resection (UTPWR) is 2005 (5). Since then, the uniportal thoracoscopy technology has been widely used for lobar or segmental lung resection (6). According to research, UTPWR is particularly well-suited for early-stage peripheral lung cancer treatment and lung tissue biopsy (7). Notably, although UTPWR has been associated with minimal surgical trauma at the operative level, it has a longer surgical incision and involves postoperative drainage tube stimulation, which causes severe pain. Additionally, the pain following UTPWR is mostly intense within 24 h post-surgery. Furthermore, the severe post-UTPWR pain could prevent patients from taking deep breaths and coughing, potentially causing postoperative pulmonary complications and affecting postoperative recovery (8).

Currently, there are no more straightforward, more effective, and safer analgesic methods for reducing post-UTPWR pain. The current standard analgesic interventions following thoracic surgery include thoracic epidural analgesia (TEA), ultrasound-guided thoracic paravertebral block (UG-TPB), and patient controlled intravenous analgesia (PCIA). Although TEA and UG-TPB can effectively reduce thoracic surgery-related postoperative pain and opioid consumption, they have high operational requirements, as well as failure and complication rates, limiting their clinical application (9, 10). Furthermore, the operation of epidural analgesia and UG-TPB is cumbersome, making them unsuitable for postoperative analgesia after UTPWR. On the other hand, PCIA, a commonly used postoperative analgesia method in clinical practice, offers the benefits of simple operation and convenient postoperative care. However, its systemic use of opioid drugs is relatively high, leading to more adverse reactions (ARs) (11).

**Figure 1:**
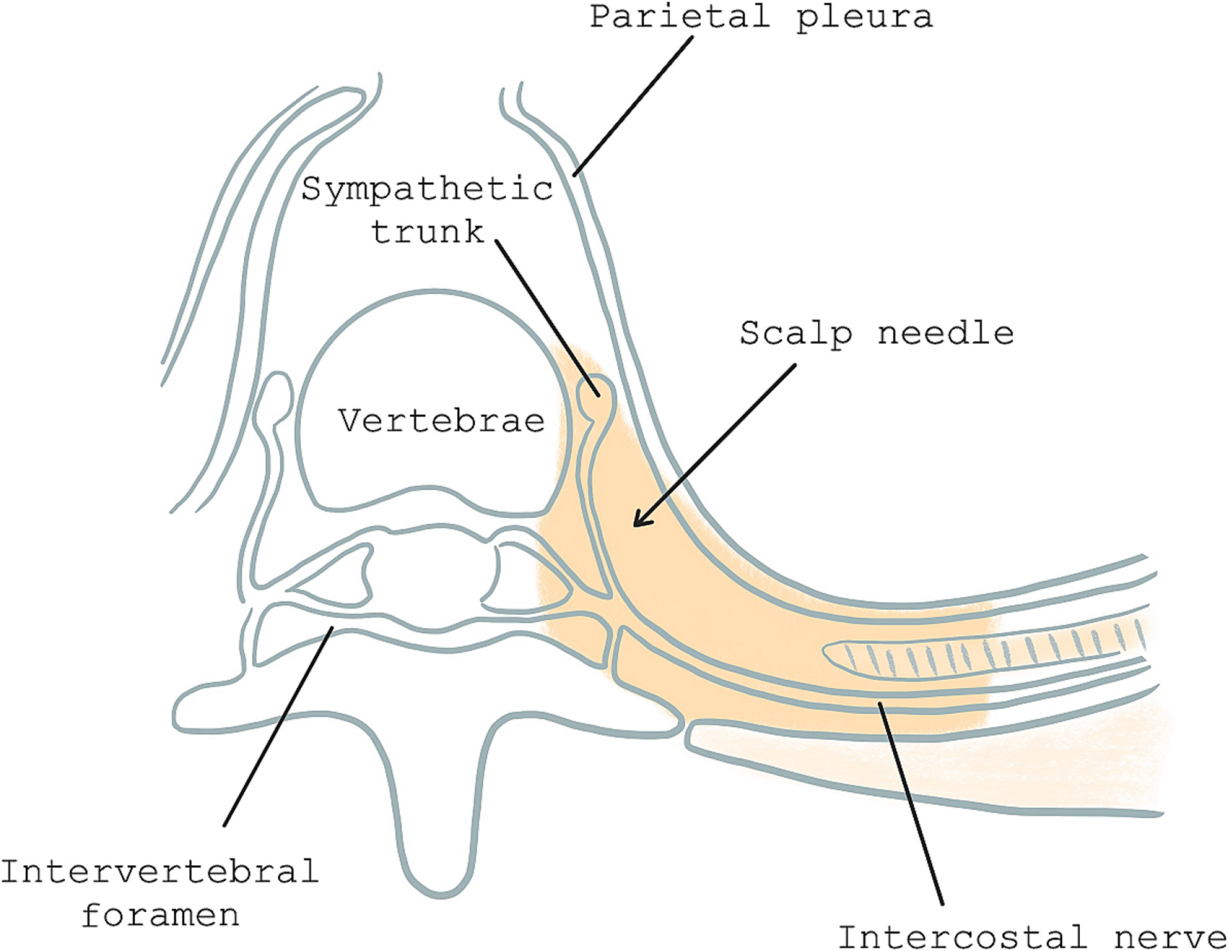
Horizontal plane of paravertebral space: the gray area represents paravertebral space and site of scalp needle puncture.

Our research team previously validated thoracoscopic-guided thoracic paravertebral block (TG-TPB), a novel thora11ic paravertebral block technique. This intervention involves the injection of local anesthetics (LAs) into the thoracic paravertebral space via the intra-thoracic approach under thoracoscopic guidance (12). Following TG-TPB, LAs diffused into the paravertebral space across multiple levels. Consequently, our research team concluded that TG-TPB was simple to operate and could effectively relieve post-UTPWR pain (Figure 1) (13). However, they used PCIA in both the observation and control groups. In this regard, it remains unclear whether TG-TPB could be used alone to reduce postoperative pain following UTPWR. This study compared the analgesic effects of TG-TPB and PCIA post-UTPWR and aimed to establish whether TG-TPB alone, when applied for analgesia post-UTPWR, could meet analgesia needs and decrease opioid consumption.

## Methods

### Study design and population

This prospective, double-blind, randomized controlled trial (RCT) was approved by the Medical Ethics Committee of the Ningbo University-Affiliated Lihuili Hospital (Grant No. KY2020PJ015) and submitted to the Chinese Clinical Trial Registry (Registration No. ChiCTR2000034726; 16/07/2020). The study protocol adhered to the ethical guidelines of the 1975 Helsinki Declaration and the Consolidated Standards of Reporting Trials (CONSORT) statement. The study began actual research and enrollment of the first patient on January 5, 2021. Between January and June 2021, 60 patients undergoing UTPWR were enrolled from the Ningbo University-affiliated Lihuili Hospital. All patients signed an informed consent form before participating in the study. The inclusion criteria were as follows: (1) patients aged 18–70 years; and (2) patients with pulmonary nodules or early-stage peripheral lung cancer and require thoracoscopic surgery. On the other hand, the exclusion criteria were as follows: (1) patients on anticoagulant medication or with coagulopathy; (2) patients who refused to undergo UTPWR; (3) patients whose intraoperative pathologies confirmed an invasive malignancy; (4) patients who were allergic to LAs; (5) patients with severe pleural adhesion; (6) patients undergoing an unplanned surgery after the surgery; (7) patients with chronic pain, especially chronic pain in the chest; (8) patients with long term use of painkillers; (9) patients whose procedure was changed to multi-hole thoracoscopy or open chest surgery; and (10) patients that requested withdrawal from the study.

### Randomization and blindness

Herein, participants were randomly allocated to two groups: P and T (1:1). We used SPSS v24.0 (IBM Corp. Armonk, NY, United States) to generate sequential numbers from 1 to 60 and prepared 60 envelopes. In the second step, we utilized SPSS’s random number generator to produce a random sequence of these 60 numbers. In the third step, we ranked the random numbers and placed the ranking results into the envelopes. In the fourth step, we assigned those with odd rankings to P group and those with even rankings to T group. In the fifth step, a envelope was randomly selected after patients were enrolled. A non-blind nurse responsible for grouping assigned patients to their respective groups based on the ranking inside the envelope. This non-blinded nurse prepared a 20 mL 0.375% ropivacaine or 0.9% sodium chloride (NaCl) solution per the grouping results. The nurse also prepared 100 mL sufentanil or 0.9% NaCl for the PCIA pump. This nurse did not participate in the follow-up study. Other surgeons and anesthesiologists involved in the aforementioned processes did not also participate in the subsequent aspects of the research. All patients, surgeons, anesthesiologists, and data collectors were unaware of the grouping. The same surgical team managed the surgical and anesthesia procedures.

### Anesthesia procedures

All surgeries were performed under total intravenous anesthesia, which was induced using 0.06 mg/kg midazolam, 0.4 μg/kg sufentanil, 1.0–1.5 mg/kg propofol, and 0.9 mg/kg rocuronium. A double-lumen bronchial tube was then inserted after rapid intravenous induction. The ventilator was connected to the bronchial tube for mechanically controlled ventilation, with tidal volumes of 6 mL/kg and 8–10 times/min (13).

### Surgical procedures

After anesthesia induction and endotracheal intubation, the patients underwent UTPWR. Specifically, a ≤4 cm incision was made at the fourth intercostal levels of the mid-axillary line. Pathological examination was then performed to confirm the nature of the tumor. The surgeon closed the chest and ended the operation if the tumor was benign or an early-stage malignancy. On the other hand, if it was an infiltrating malignant tumor, the surgeon performed lobectomy or segmental resection and cleaned the hilar lymph nodes. Such cases were to be excluded from this study (13). An Fr16 (5.33 mm) thoracic drainage tube was left in place at the end of the surgery and was later removed 24 h post-surgery.

### Analgesia management

**Figure 2:**
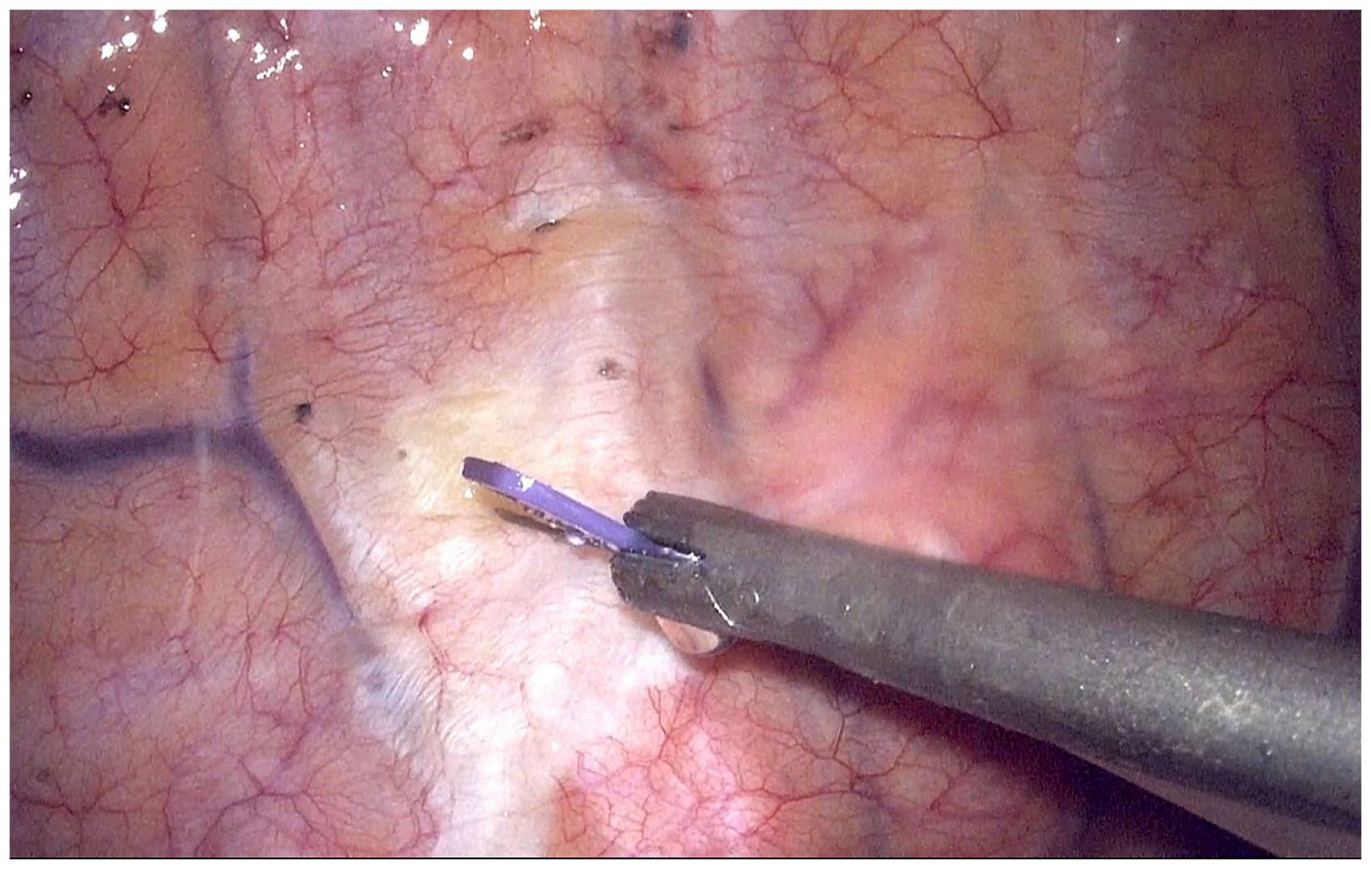
Thoracoscopic guided thoracic paravertebral block at fourth intercostal levels. The white raised area indicates the diffusion of local anesthetics in the paravertebral space.

Both groups received 0.2 μg/kg sufentanil 30 min before the end of surgery. Furthermore, T group patients received TG-TPB before the chest was closed. Under thoracoscopic guidance, a 25G (0.5 mm) needle with an infusion tube was vertically inserted into the thoracic paravertebral space at the fourth intercostal plane and 0.2 cm lateral to the sympathetic chain. The puncture depth was ~0.5 cm below the parietal pleura. Subsequently, 20 mL 0.375% ropivacaine was injected (Figure 2). Conversely, P group patients received TG-TPB with 20 mL 0.9% NaCl.

At the end of the procedure, P group patients were connected to a PCIA pump containing 100 ug sufentanil and 5 mg tropisetron diluted to a 100 mL solution with 0.9% NaCl. On the other hand, T group patients were connected to a PCIA pump containing 100 mL 0.9% NaCl and 5 mg tropisetron. The PCIA execution parameters were a 2 mL/h continuous dose, a 2 mL/time bolus dose, and a 15-min lock.

The patients were administered rescue analgesia (2 mg morphine injected intravenously) if the pain did not relieved 15 min after PCIA compression.

### Data collection

The primary outcomes included the Visual Analogue Scale (VAS) scores (during rest and coughing) recorded at 2, 6, 12, 24, and 48 h postoperatively and total sufentanil consumption within 24 h postoperatively. Total sufentanil consumption within 24 h postoperatively encompassed sufentanil consumption during PCIA administration and the morphine dose for rescue analgesia converted to the equivalent dose of sufentanil.

On the other hand, the secondary outcomes included the number of PCIA presses, rescue analgesia interventions, and AR incidences recorded within 24 h postoperatively. The time points of PCIA presses and rescue analgesia were also recorded. The number of additional analgesia in each time period was recorded (number of PCIA presses and rescue analgesia). Key ARs included nausea, vomiting, drowsiness, pruritus, respiratory depression, and atelectasis. The time taken to remove the thoracic drainage tube and bleeding incidences at the puncture point of T group patients were also recorded. Furthermore, the 15-item Quality of Recovery Scale (QoR-15) scores were recorded 24 h postoperatively.

### Sample size

A Pilot test with 10 patients revealed that the mean (standard deviation) postoperative VAS scores at 6 h were 2.8 (0.6) and 3.4 (0.6) in the T and P groups, respectively. Based on the power and alpha values of 0.8 and 0.05, respectively, the MedSci Sample Size Tools revealed that the minimum number of cases per group was 17. However, considering the 20% probability of missing or withdrawn data, ≥ 21 patients were recruited for each group.

### Statistical analysis

Statistical analyses were performed using SPSS v24.0 (IBM Corp. Armonk, NY, United States). Normally distributed quantitative data were expressed as mean ± standard deviation (SD). Inter-group differences were evaluated using the *t*-test. On the other hand, intra-group comparisons were conducted using repeated-measures analysis of variance (ANOVA). The Bonferroni correction was chosen to control the type I error. Non-normally distributed data were presented as medians and interquartile ranges (IQRs) and compared using the Kruskal–Wallis *H* test. Inter-group differences in the counting data were evaluated using Fisher’s exact test or the chi-square (*χ*^2^) test. In all analyses, results with *p* < 0.05 were considered statistically significant.

## Results

### Patients’ baseline and intraoperative data

**Figure 3:**
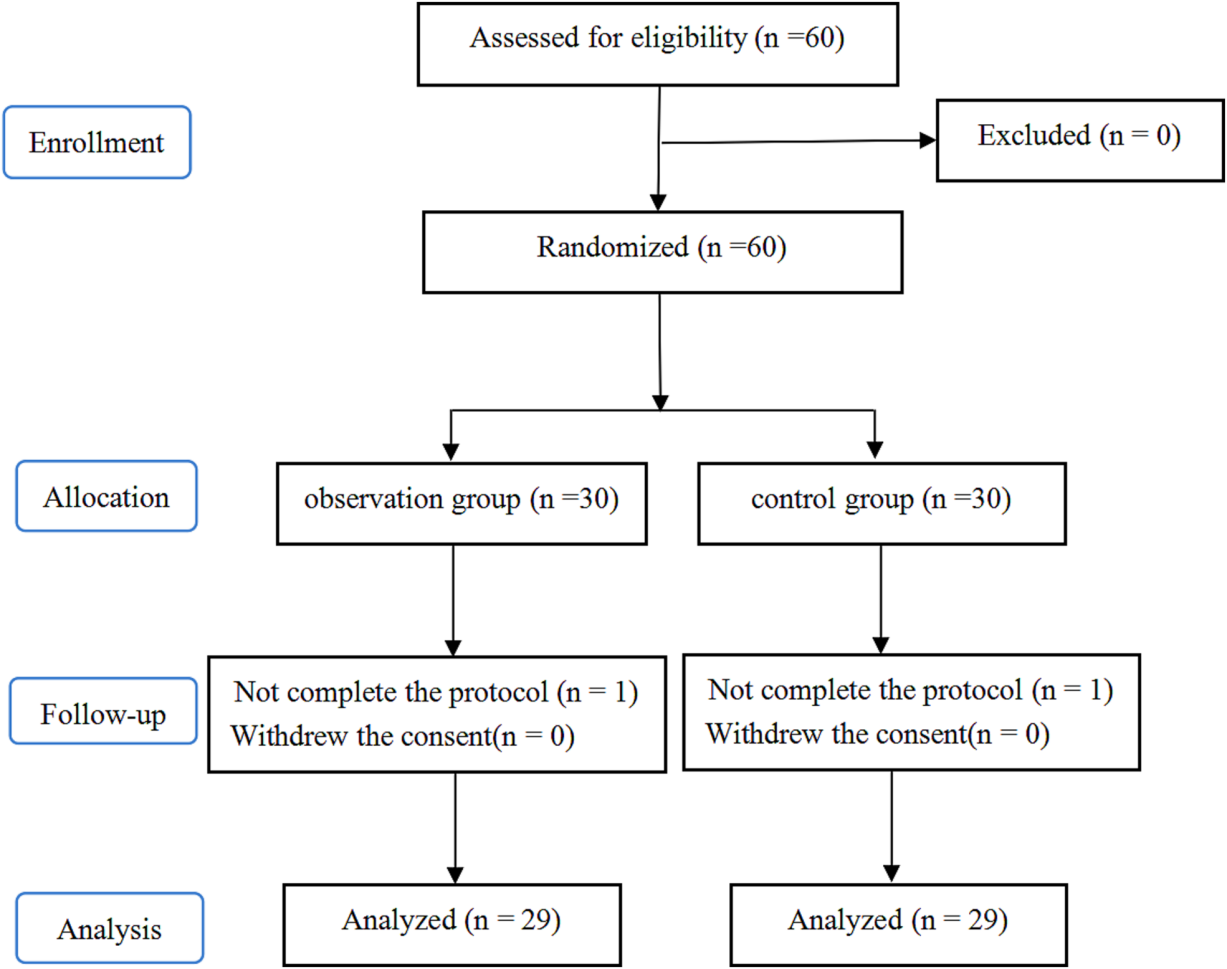
Flow diagram of study.

This study involved 60 patients. Among them, one patient in the P group was lost to follow-up, and one patient in the T group had an intraoperative pathological indication of an infiltrating tumor and underwent lobectomy and lymph node dissection instead. Consequently, 58 participants were included in the final analysis. Figure 3 shows the CONSORT flow diagram. The two groups showed no significant differences in baseline and intraoperative data (*p* > 0.05, Table 1).

**Table 1:** The baseline data and intraoperative data of patients.

Baseline and intraoperative data	P group (*n* = 29)	T group (*n* = 29)	*p*-value
Number of patients	29	29	
Surgical site			0.793
Left	15 (51.7%)	14 (48.3%)	
Right	14 (48.3%)	15 (51.7%)	
Gender			0.599
Male	16 (55.2%)	14 (48.3%)	
Female	13 (44.8%)	15 (51.7%)	
Age (years)	44.6 ± 15.0	43.9 ± 12.4	0.845
Age stratification			0.942
18–39 (years)	11	11	
40–54 (years)	11	12	
55–70 (years)	7	6	
Weight (kg)	62.7 ± 8.8	59.0 ± 9.2	0.128
BMI	22.4 ± 1.8	22.6 ± 2.2	0.618
Surgery time (min)	21.8 ± 4.3	23.4 ± 5.2	0.191

BMI, body mass index. Data are presented as mean ± standard deviation or number (%).

### Primary outcomes

**Figure 4:**
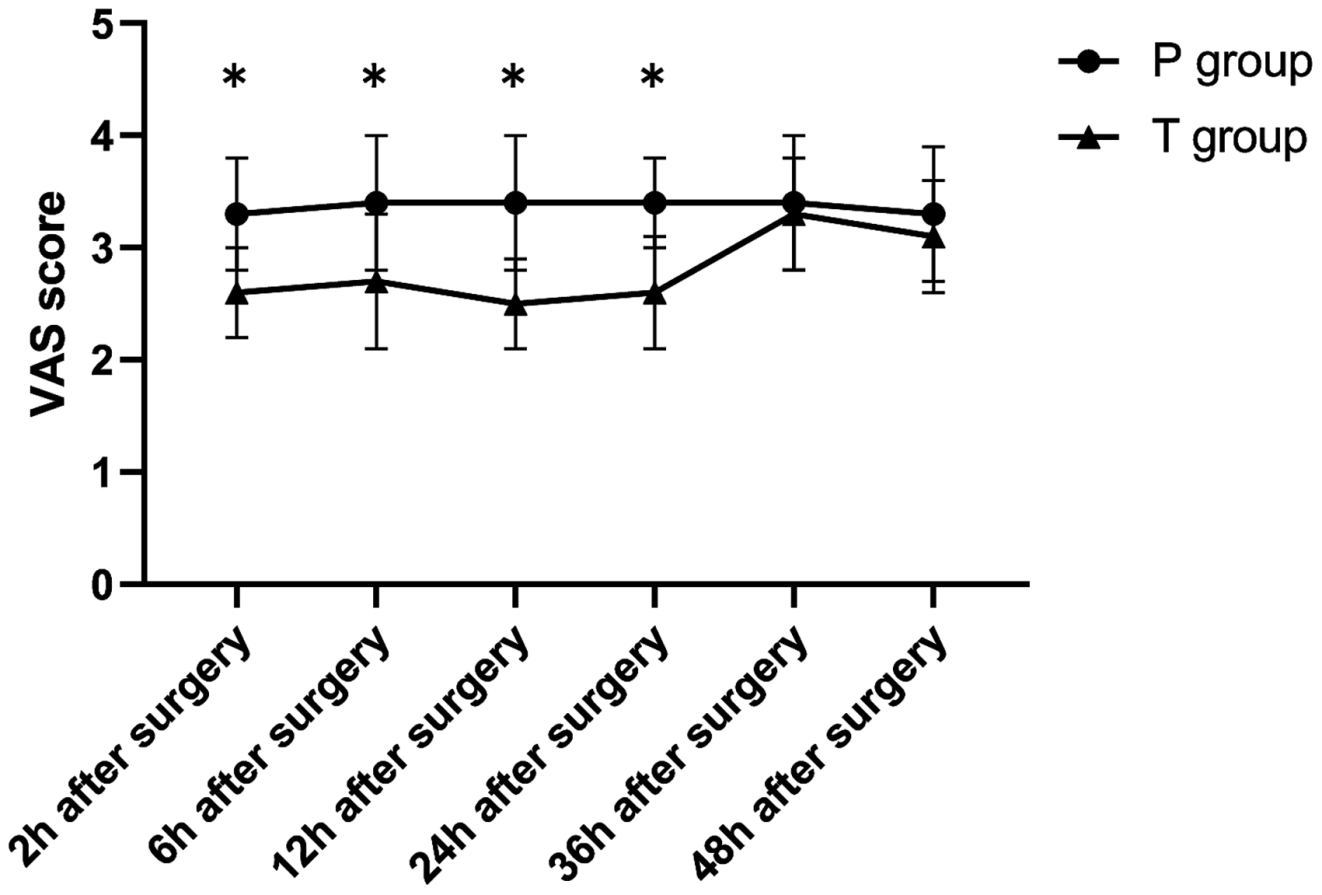
The VAS score (during rest) after surgery. ^*^p < 0.001 T group vs. P group.

**Figure 5:**
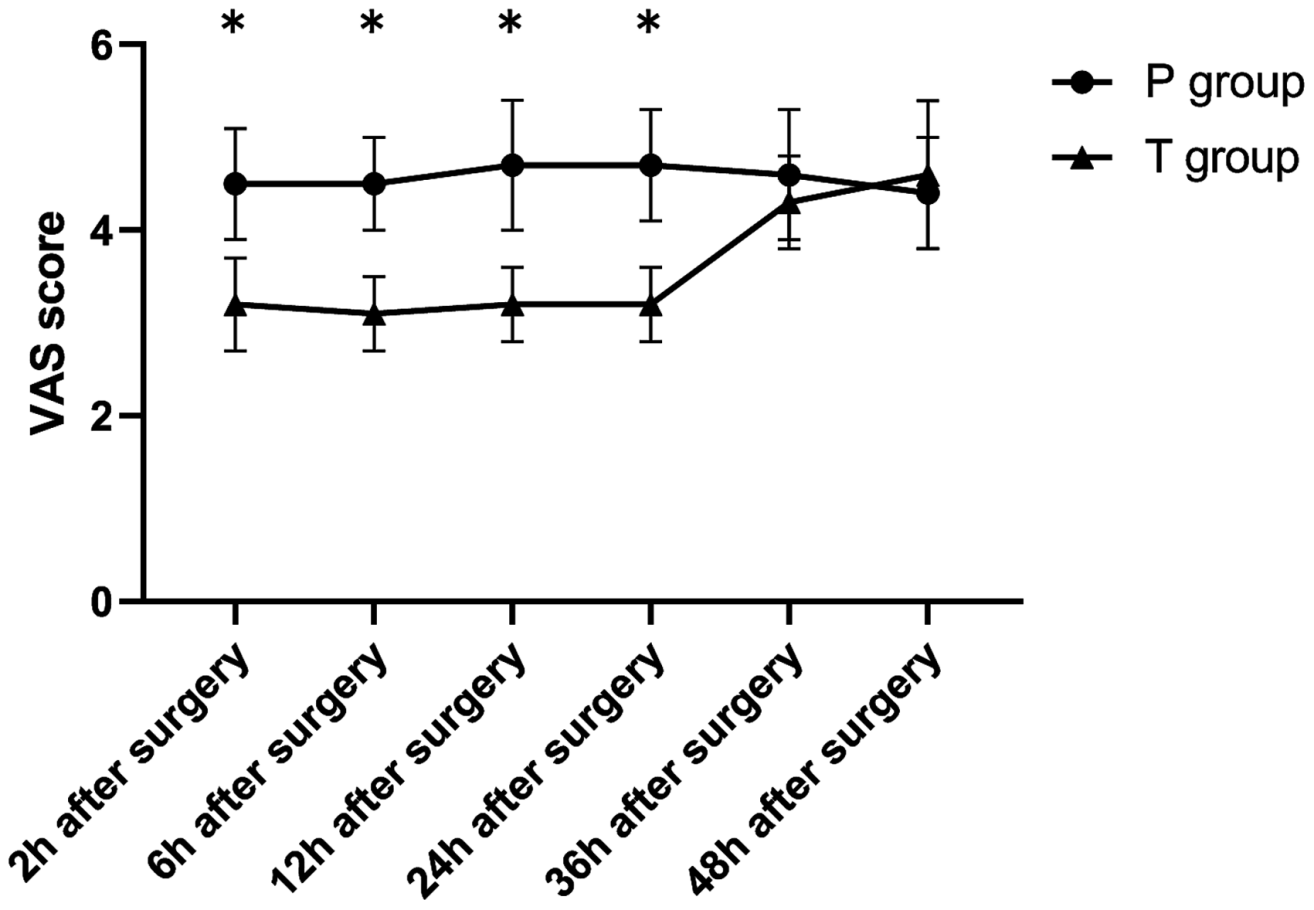
The VAS score (while coughing) after surgery. ^*^p < 0.001 T group vs. P group.

The T group showed significantly lower VAS scores during rest and coughing at 2, 6, 12, and 24 h postoperatively compared to at 36 and 48 h postoperatively (*p* < 0.001). On the other hand, the P group showed no significant differences in VAS scores (rest and coughing) across various time points (*p* > 0.05). Notably, compared to the P group, the T group showed significantly lower VAS scores (rest and coughing) at 2, 6, 12, and 24 h postoperatively (*p* < 0.001). Furthermore, the two groups showed no statistically significant differences in VAS scores at 36 and 48 h postoperatively (*p* > 0.05) (Table 2 and Figures 4, 5). Moreover, total sufentanil consumption within 24 h postoperatively was lower in the T group than in the P group [0 (0, 0) vs. 58.8 (60.1, 64.2) μg, *z* = −6.786, *p* < 0.001].

**Table 2:** The VAS score after surgery.

Scoring status	Time points	P group (*n* = 30)	T group (*n* = 30)	*p*-value
During rest	2 h after surgery	3.3 ± 0.5	2.6 ± 0.4	<0.001
	6 h after surgery	3.4 ± 0.6	2.7 ± 0.6	<0.001
	12 h after surgery	3.4 ± 0.6	2.5 ± 0.4	<0.001
	24 h after surgery	3.4 ± 0.4	2.6 ± 0.5	<0.001
	36 h after surgery	3.4 ± 0.6	3.3 ± 0.5	0.436
	48 h after surgery	3.3 ± 0.6	3.1 ± 0.5	0.122
	*F* value	0.188	12.192	
	*p*-value	0.967	<0.001	
While coughing	2 h after surgery	4.5 ± 0.6	3.2 ± 0.5	<0.001
	6 h after surgery	4.5 ± 0.5	3.1 ± 0.4	<0.001
	12 h after surgery	4.7 ± 0.7	3.2 ± 0.4	<0.001
	24 h after surgery	4.7 ± 0.6	3.2 ± 0.4	<0.001
	36 h after surgery	4.6 ± 0.7	4.3 ± 0.5	0.103
	48 h after surgery	4.4 ± 0.6	4.6 ± 0.8	0.156
	*F* value	1.165	49.672	
	*p*-value	0.330	<0.001	

Data are presented as mean ± standard deviation.

### Secondary outcomes

The T group showed a lower number of PCIA presses within 24 h postoperatively than the P group [1 (1, 2) vs. 6 (6, 7), *z* = −6.624, *p* < 0.001]. Five T group patients were administered one rescue analgesia intervention each. Furthermore, the T group had a significantly lower number of rescue analgesia interventions within 24 h postoperatively than the P group [0 (0, 0) vs. 2 (1, 3.5) times, *z* = −5.818, *p* < 0.001]. The number of additional analgesia during each 6 h period within 24 h postoperatively were significantly lower in the T group than in the P group [0 (0, 0) vs. 3 (2, 4), *z* = −6.882, *p* < 0.001; 0 (0, 0) vs. 3 (2, 4), *z* = −6.832, *p* < 0.001; 0 (0, 1) vs. 2 (1, 2), *z* = −5.416, *p* < 0.001; 0 (0, 1) vs. 1 (1, 1), *z* = −2.139, *p* = 0.032] (Table 3).

**Table 3:** Additional analgesia in different time periods within 24 h after surgery.

Time period	P group	T group	*Z*	*p*-value
0–6 h	3 (2, 4)	0 (0, 0)	−6.882	<0.001
7–12 h	3 (2, 4)	0 (0, 0)	−6.832	<0.001
13–18 h	2 (1, 2)	0 (0, 1)	−5.416	<0.001
19–24 h	1 (1, 1)	0 (0, 1)	−2.139	0.032

Data are presented as medians and interquartile ranges.

Compared to the P group, the T group had lower incidences of nausea and vomiting (*p* < 0.05). Furthermore, the two groups showed no statistical differences in the incidences of drowsiness, pruritus, respiratory depression, and atelectasis (Table 4). Notably, two cases of puncture site bleeding occurred in the T group. Additionally, the two groups showed no differences in the removal time of thoracic drainage tubes (22.2 ± 2.2 vs. 22.7 ± 2.4, *p* = 0.349).

**Table 4:** The incidence of adverse reactions after surgery.

Adverse reactions	P group (*n* = 29) (%)	T group (*n* = 29) (%)	*p*-value
Nausea	13 (44.8%)	4 (13.8%)	0.009
Vomit	10 (34.5%)	3 (10.3%)	0.028
Pruritus	3 (13.3%)	0 (0.0%)	0.236
Respiratory depression	3 (13.3%)	0 (0.0%)	0.236
Drowsiness	4 (10.0%)	0 (0.0%)	0.120
Atelectasis	3 (10.3%)	1 (6.9%)	0.604

Data are presented as number (%).

The two groups also showed no statistical differences in the pre-operative QoR-15 scores (120.4 ± 15.0 vs. 118.7 ± 8.6, *p* = 0.600). Nonetheless, the T group had a higher postoperative QoR-15 score at 24 h than the P group (90.5 ± 7.3 vs. 76.6 ± 6.2, *p* < 0.001) (Table 5).

**Table 5:** The QoR-15 of each group.

Time points	P group (*n* = 29)	T group (*n* = 29)	*p*-value
Preoperation	120.4 ± 15.0	118.7 ± 8.6	0.600
Postoperation	76.6 ± 6.2	90.5 ± 7.3	<0.001
*p*-value	<0.001	<0.001	

Data are presented as mean ± standard deviation.

## Discussion

Herein, we discovered that TG-TPB exerted a better analgesic effect than PCIA within 24 h post-UTPWR, with less opioid consumption and AR incidences and significantly better postoperative recovery quality.

Conventional thoracotomy has been established to cause severe trauma and pain, affecting postoperative rehabilitation (14). Consequently, thoracoscopic minimally invasive surgical approaches have recently gained significant traction as alternatives to conventional thoracotomy (15). Among them is UTPWR, which is particularly useful in treating pulmonary bullae, pneumothorax, or pulmonary nodules, especially when considering carcinoma *in situ* (16). Compared to traditional multi-hole thoracoscopic surgery, UTPWR offers the benefits of fewer incisions, shorter surgical durations, less postoperative pain, and faster recovery (4). However, it is noteworthy that surgical staff often overlook the significance of postoperative pain relief precisely due to the aforementioned advantages of UTPWR. Furthermore, in UTPWR, the surgical incision is extended to 3–4 cm, further exacerbating the damage to the intercostal nerve. Additionally, UTPWR involves the stimulation of the postoperative thoracic drainage tube, increasing the persistence of the postoperative pain and thus delaying recovery post-surgery. Patients undergoing thoracic surgery also experience decreased postoperative lung function reserve, severe postoperative pain, and challenges in deep breathing and coughing, potentially leading to additional postoperative pulmonary complications. Therefore, adequate postoperative analgesia is critical for faster patient rehabilitation and ambulation, potentially enhancing respiratory function, reducing the incidence of pulmonary complications, and accelerating postoperative recovery (17).

Presently, TEA, UG-TPB, and PCIA are the most commonly used forms of postoperative analgesia following thoracic surgery. Among them, TEA is considered the gold standard for postoperative analgesia in thoracic surgery, particularly for procedures involving considerable trauma, such as thoracotomy. However, TEA has many drawbacks, such as puncture failure, epidural perforation, epidural hematoma or abscess, and nerve damage (9). On the other hand, the thoracic paravertebral space, where UG-TPB is often performed, is anatomically triangular and contains the intercostal nerves and sympathetic nerve chains originating from the intervertebral foramen. The thoracic paravertebral nerve blockade is mainly achieved via ultrasound-guided single dose blocks or inserting a catheter into the extra-pleural paravertebral space for a continuous block via ultrasound-guided percutaneous punctures. Following UG-TPB, the LAs spread in the paravertebral space across 4–8 segments, leading to the simultaneous blockade of all the nerves in the paravertebral space. Notably, paravertebral nerve blocks can effectively reduce incision and visceral pain (18, 19); hence, they have been widely used in clinical settings (20). However, they also have some drawbacks, such as higher medical and resource requirements, limited operation space, and a low success rate. Finally, although PCIA, a commonly used clinical postoperative analgesic approach, offers the benefits of simple operation and precise analgesic effects, its systemic use of opioid drugs is relatively high, and it has also been associated with various ARs such as postoperative nausea, vomiting, drowsiness, and respiratory depression (11).

Since UTPWR has a short surgical duration and minimal surgical trauma, the resulting postoperative pain is mainly concentrated within 24 h post-surgery. Compared to other types of thoracic surgery, UTPWR has shown a significant improvement in the degree and duration of postoperative pain (21). Given that TEA and UG-TPB are cumbersome and with many complications and a high failure rate, they are not considered ideal for postoperative analgesia post-UTPWR. This leaves PCIA as the primary postoperative analgesia technique following UTPWR. Nonetheless, the many ARs resulting from opioid use, which is common in PCIA, remain a challenge (11, 22). Fortunately, with the application of the concept of accelerated rehabilitation surgery and the multimodal opioid-sparing analgesic strategy, the perioperative use of few or no opioids is gaining traction (23, 24).

Notably, TG-TPB, a novel TPB technique, involves the thoracoscopy-guided puncturing of the blocking needle through the parietal pleura before directly injecting LAs into the paravertebral space. Our previous findings validated the effectiveness and safety of TG-TPB. Specifically, in addition to its simplicity and convenience, we found that TG-TPB could significantly reduce postoperative pain and enhance recovery after minimally invasive esophageal cancer surgery (12). In another study, we discovered that compared to UG-TPB, TG-TPB was a simpler surgical intervention, with shorter surgical time and superior analgesic effects (25). Our other study revealed that TG-TPB paired with PCIA reduced pain and ARs and accelerated postoperative recovery in patients that underwent UTPWR (13). Based on these findings, we sought to establish whether TG-TPB alone could effectively reduce postoperative pain after UTPWR. Therefore, we compared the postoperative analgesic effects, opioid consumption levels, number of complications, and postoperative recovery quality between surgical patients treated with TG-TPB and PCIA. Besides assessing its postoperative analgesic effects, we also sought to establish whether TG-TPB alone could reduce opioid consumption during the perioperative period.

Herein, consistent with previous studies (12, 13, 25), which demonstrated the successful implementation of the thoracic paravertebral block, LAs were effectively diffused in the paravertebral space across 4–8 vertebral segments under thoracoscopy. Compared to the P group, the T group, at each time point within 24 h post-surgery, showed lower VAS scores, sufentanil consumption levels, number of PCIA presses, and number of rescue analgesia interventions, indicating that TG-TPB exerted better analgesic effects than PCIA and further proving the former’s effectiveness and feasibility. Furthermore, LAs diffused in the paravertebral space of several segments, covering the entire range of the surgical and chest wall incision, thus significantly alleviating postoperative pain. Additionally, the duration of a single thoracic paravertebral block correlated with LA concentration, typically ranging between 12 and 24 h (26). In the T group, the VAS scores at each time point within 24 h postoperatively were markedly lower than those at 36 and 48 h postoperatively, further confirming the efficacy and feasibility of TG-TPB. Although PCIA still exerted analgesic effects 24 h post-surgery in the P group, there were no statistically significant differences between the two groups in VAS scores at 36 and 48 h postoperatively. This phenomenon could be attributed to the fact that UTPWR has less trauma, and its postoperative pain is mostly intense within 24 h post-surgery. Therefore, a single TG-TPB dose can fully meet the postoperative analgesia needs post-UTPWR.

Total sufentanil consumption within 24 h postoperatively encompassed consumption during PCIA administration and the morphine dose for rescue analgesia converted to a sufentanil equivalent dose. Since the PCIA pump of T group patients only contained 0.9% NaCl, the postoperative consumption of opioid drugs among T group patients was only for rescue analgesia. In addition, the T group had a significantly lower number of rescue analgesia interventions within 24 h postoperatively than the P group. This finding suggests that TG-TPB can effectively alleviate postoperative pain within 24 h after UTPWR and achieve postoperative analgesia with little or no opioid drug consumption. In order to more accurately analyze the analgesic effect and rescue analgesia within 24 h postoperatively, this study divided 24 h postoperatively into four time periods every 6 h for comparison. The number of rescue analgesia during each 6 h period within 24 h postoperatively were significantly lower in the T group than in the P group. This suggested that the TG-TPB block was successful in the T group, while the TG-TPB block in the P group was unsuccessful because only saline was used. In particular, the T group did not give any form of additional analgesia during the 0–6 h and 7–12 h periods, which was significantly lower than that in the P group. Although five patients in T group received rescue analgesia intervention within 13-24 h after surgery, the number of rescue analgesia in T group was also significantly lower than that in P group during 13-18 h and 19-24 h. This suggest that the analgesic effect of TG-TPB may attenuate in the postoperative 13-24 h stage, but it still has a good analgesic effect. TG-TPB can meet the postoperative analgesia needs after 24 h of UTPWR and can achieve no or less opioid consumption for postoperative analgesia. We administered T group patients with a PCIA pump containing only 0.9% NaCl to achieve a double-blind study design and make the research results more reliable. Furthermore, PCIA pumps containing only physiological saline may have a potential placebo effect. To minimize the potential placebo effect of PCIA, we told all patients in both groups that they would be connected to the PCIA pump after surgery, while all patients were not aware of the drug allocation in the PCIA pump.

Herein, the T group had a lower incidence of nausea and vomiting than the P group, attributable to the fact that P group patients used a large amount of opioid drugs for pain relief post-surgery, whereas only a few T group patients used opioid drugs. This finding further suggests that TG-TPB can achieve postoperative analgesia post-UPTWR with little or no opioid drug use. Furthermore, no atelectasis occurred in either group, potentially due to less lung tissue removed, a shorter surgical duration, and a shorter one lung ventilation time, among other factors. Additionally, two cases of puncture site bleeding occurred in the T group. To stop bleeding at the puncture point, a gentle press with gauze is required (24). Moreover, thoracoscopy has an amplification function, through which blood vessels could be avoided as much as possible during the blocking operation, thus reducing bleeding incidences. The entire operation of TG-TPB is performed under thoracoscopic vision, which also helps to reduce or avoid complications of TG-TPB operation. This also fully reflects the security of TG-TPB.

The QoR-15 scale is a simplified version of the 40-item Quality of Recovery Scale. It is a patient-centered self-rating scale for evaluating early postoperative recovery quality (27–29). The QoR-15 scale has excellent validity, reliability, responsiveness, clinical acceptability, and feasibility, making it a valuable measure for assessing patient’s early health status, quality of life, and efficacy of anesthesia after surgery. Herein, the T group showed higher QoR-15 scores than the P group at 24 h postoperatively, implying that T group patients had a higher postoperative recovery quality than P group patients. This outcome could be attributed to the lighter postoperative pain and fewer ARs in the observation group. Notably, severe or persistent pain, nausea, and vomiting, among other ARs could also lead to anxiety and depression in patients, causing both physical and psychological complications, which may directly affect their early postoperative recovery quality.

This study had some limitations. First, we did not examine the sensory block level of TG-TPB. Second, we only observed the pain within 48 h after surgery, and did not observe pain scores at 48–72 h or longer. Third, we did not consider the influence of any stratification factors (age, gender, baseline pain tolerance, anxiety, and pain perception) on the grouping balance. Fourth, we did not evaluate the pain levels during thoracic drainage tube removal. Fifth, only specific patient populations such as pulmonary nodules were selected for this study, and patients with lobes or segment resection were not considered. They are also what we should consider in our subsequent study.

## Conclusion

Compared to PCIA, TG-TPB exerted superior analgesic effects, with less opioid consumption, fewer ARs, and a more significant improvement in prognostic quality within 24 h post-UTPWR. In other words, TG-TPB is a simple and convenient analgesia induction approach which can fully meet analgesia needs post-UTPWR, with little or no opioid drug use.

## Data Availability

The original contributions presented in the study are included in the article/supplementary material, further inquiries can be directed to the corresponding authors.

## References

[1] Leschber G. Video-assisted thoracic surgery: a global development. Chirurg. (2018) 89:185–90. doi: 10.1007/s00104-018-0592-7, PMID: 29468327

[2] Forster C Gonzalez M. Uniportal video-assisted thoracic surgery segmentectomy: a promising new development for thoracic surgery. Transl Lung Cancer Res. (2023) 12:1152–5. doi: 10.21037/tlcr-23-197, PMID: 37425413 PMC10326782

[3] Migliore M. Efficacy and safety of single-trocar technique for minimally invasive surgery of the chest in the treatment of noncomplex pleural disease. J Thorac Cardiovasc Surg. (2003) 126:1618–23. doi: 10.1016/S0022-5223(03)00592-0, PMID: 14666042

[4] Mizukami Y Takahashi Y Adachi H. Single-port vs. conventional three-port video-assisted thoracoscopic pulmonary wedge resection: comparison of postoperative pain and surgical costs. Ann Thorac Cardiovasc Surg. (2021) 27:91–6. doi: 10.5761/atcs.oa.20-00142, PMID: 32999140 PMC8058539

[5] Rocco G Martin-Ucar A Passera E. Uniportal VATS wedge pulmonary resections. Ann Thorac Surg. (2004) 77:726–8. doi: 10.1016/S0003-4975(03)01219-0, PMID: 14759479

[6] Wang L Ge L Song S Ren Y. Clinical applications of minimally invasive uniportal video-assisted thoracic surgery. J Cancer Res Clin Oncol. (2023) 149:10235–9. doi: 10.1007/s00432-023-04920-x, PMID: 37269347 PMC11797689

[7] Ahn S Moon Y. Uniportal video-assisted thoracoscopic surgery without drainage-tube placement for pulmonary wedge resection: a single-center retrospective study. J Cardiothorac Surg. (2022) 17:317. doi: 10.1186/s13019-022-02053-9, PMID: 36527034 PMC9758863

[8] Poupore AK Stem M Molena D Lidor AO. Incidence, reasons, and risk factors for read mission after surgery for benign distal esophageal disease. Surgery. (2016) 160:599–606. doi: 10.1016/j.surg.2016.04.037, PMID: 27365228

[9] Slinchenkova K Lee K Choudhury S Sundarapandiyan D Gritsenko K. A review of the paravertebral block: benefits and complications. Curr Pain Headache Rep. (2023) 27:203–8. doi: 10.1007/s11916-023-01118-1, PMID: 37294514

[10] Wu Z Wang Q Wu C Wu C Yu H Chen C. Paravertebral versus epidural anesthesia for video-assisted thoracic surgery: a randomized trial. Ann Thorac Surg. (2023) 116:1006–12. doi: 10.1016/j.athoracsur.2023.07.038, PMID: 37573993

[11] Yan G Chen J Yang G Duan G Du Z Yu Z. Effects of patient-controlled analgesia with hydromorphone or sufentanil on postoperative pulmonary complications in patients undergoing thoracic surgery: a quasi-experimental study. BMC Anesthesiol. (2018) 18:192. doi: 10.1186/s12871-018-0657-7, PMID: 30567490 PMC6300916

[12] Hu L Xu X Shen W He J. Feasibility and effectiveness of multi-injection thoracic paravertebral block via the intrathoracic approach for analgesia after thoracoscopic-laparoscopic esophagectomy. Esophagus. (2021) 18:513–21. doi: 10.1007/s10388-020-00807-9, PMID: 33403428 PMC8172493

[13] Hu L Xu X Tian H He J. Effect of single-injection thoracic paravertebral block via the intrathoracic approach for analgesia after single-port video-assisted thoracoscopic lung wedge resection: a randomized controlled trial. Pain Ther. (2021) 10:433–42. doi: 10.1007/s40122-020-00231-y, PMID: 33420979 PMC8119565

[14] Bayman EO Curatolo M Rahman S. Brennan TJ.AAAPT diagnostic criteria for acute thoracic surgery pain. J Pain. (2021) 22:892–904. doi: 10.1016/j.jpain.2021.03.148, PMID: 33848682

[15] Draeger TB Gibson VR Fernandes G Andaz SK. Enhanced recovery after thoracic surgery (ERATS). Heart Lung Circ. (2021) 30:1251–5. doi: 10.1016/j.hlc.2021.01.014 33726996

[16] Kang DK Min HK Jun HJ Hwang YH Kang MK. Early outcomes of single-port video-assisted thoracic surgery for primary spontaneous pneumothorax. Korean J Thorac Cardiovasc Surg. (2014) 47:384–8. doi: 10.5090/kjtcs.2014.47.4.384, PMID: 25207248 PMC4157502

[17] Deana C Vetrugno L Bignami E Bassi F. Peri-operative approach to esophagectomy: a narrative review from the anesthesiological standpoint. J Thorac Dis. (2021) 13:6037–51. doi: 10.21037/jtd-21-940, PMID: 34795950 PMC8575828

[18] Jin L Yao R Heng L Pang B Sun FG Shen Y. Ultrasound-guided continuous thoracic paravertebral block alleviates postoperative delirium in elderly patients undergoing esophagectomy: a randomized controlled trial. Medicine. (2020) 99:e19896. doi: 10.1097/MD.0000000000019896, PMID: 32332664 PMC7440095

[19] Kosiński S Fryźlewicz E Wiłkojć M Ćmiel A Zieliński M. Comparison of continuous epidural block and continuous paravertebral block in postoperative analgaesia after video-assisted thoracoscopic surgery lobectomy: a randomised, non-inferiority trial. Anaesthesiol Intensive Ther. (2016) 48:280–7. doi: 10.5603/AIT.2016.0059, PMID: 28000203

[20] Wang Q Wei S Li S Yu J Zhang G Ni C. Comparison of the analgesic effect of ultrasound-guided paravertebral block and ultrasound-guided retrolaminar block in uniportal video-assisted thoracoscopic surgery: a prospective, randomized study. BMC Cancer. (2021) 21:1229. doi: 10.1186/s12885-021-08938-7, PMID: 34784889 PMC8594110

[21] Tiberi M Andolfi M Salati M Roncon A Guiducci GM Falcetta S. Impact of enhanced pathway of care in uniportal video-assisted thoracoscopic surgery. Updat Surg. (2022) 74:1097–103. doi: 10.1007/s13304-021-01217-x, PMID: 35013903

[22] Nakai A Nakada T Okamoto S Takahashi Y Sakakura N Nakada J. Risk factors for postoperative nausea and vomiting after thoracoscopic pulmonary wedge resection: pitfalls of an increased fentanyl dose. J Thorac Dis. (2021) 13:3489–96. doi: 10.21037/jtd-21-296, PMID: 34277044 PMC8264675

[23] Semenkovich TR Hudson JL Subramanian M Kozower BD. Enhanced recovery after surgery (ERAS) in thoracic surgery. Semin Thorac Cardiovasc Surg. (2018) 30:342–9. doi: 10.1053/j.semtcvs.2018.06.001, PMID: 29940227

[24] Joshi GP. Regional analgesia as the core component of multimodal analgesia technique: current controversies and future directions. J Clin Anesth. (2024) 92:111227. doi: 10.1016/j.jclinane.2023.111227, PMID: 37553267

[25] Xu X Xie YX Zhang M Du JH He JX Hu LH. Comparison of thoracoscopy-guided thoracic paravertebral block and ultrasound-guided thoracic paravertebral block in postoperative analgesia of thoracoscopic lung cancer radical surgery: a randomized controlled trial. Pain Ther. (2024) 13:577–88. doi: 10.1007/s40122-024-00593-7, PMID: 38592611 PMC11111614

[26] Krediet AC Moayeri N van Geffen GJ Bruhn J Renes S Bigeleisen PE. Different approaches to ultrasound-guided thoracic paravertebral block: an illustrated review. Anesthesiology. (2015) 123:459–74. doi: 10.1097/ALN.0000000000000747 26083767

[27] Rosato R Palazzo V Borghi F Camanni M Puppo A Delpiano EM. Factor structure of post-operative Quality of Recovery questionnaire (QoR-15): an Italian adaptation and validation. Front Psychol. (2022) 13:1096579. doi: 10.3389/fpsyg.2022.1096579, PMID: 36817374 PMC9936892

[28] Kara U Şimşek F Kamburoğlu H Özhan MÖ Alakuş Ü İnce ME. Linguistic validation of a widely used recovery score: Quality of Recovery-15 (QoR-15). Turk J Med Sci. (2022) 52:427–35. doi: 10.55730/1300-0144.5330, PMID: 36161615 PMC10381226

[29] Campfort M Cayla C Lasocki S Rineau E Léger M. Early quality of recovery according to QoR-15 score is associated with one-month postoperative complications after elective surgery. J Clin Anesth. (2022) 78:110638. doi: 10.1016/j.jclinane.2021.110638, PMID: 35033845

